# Predictive Model of Dysphagia and Brain Lesion-Symptom Mapping in Acute Ischemic Stroke

**DOI:** 10.3389/fnagi.2021.753364

**Published:** 2021-10-20

**Authors:** Lulu Zhang, Xiang Tang, Can Wang, Dongxue Ding, Juehua Zhu, Yun Zhou, Shanshan Diao, Yan Kong, Xiuying Cai, Cuiping Li, Ye Yao, Qi Fang

**Affiliations:** ^1^Department of Neurology, First Affiliated Hospital of Soochow University, Suzhou, China; ^2^Shanghai Zhiyu Software Technology Co., Ltd., Shanghai, China; ^3^Department of Biostatistics, School of Public Health, Fudan University, Shanghai, China; ^4^National Clinical Research Center for Aging and Medicine, Huashan Hospital, Fudan University, Shanghai, China; ^5^Key Laboratory of Public Health Safety of Ministry of Education, Fudan University, Shanghai, China

**Keywords:** post-stroke dysphagia, MRI, water-swallowing test, volume-viscosity swallow test, voxel-based lesion-symptom mapping

## Abstract

**Background and purpose:** Early recognition and management of post-stroke dysphagia (PSD) based on MRI may reduce the incidence of complications. Combining clinical symptoms with applications of MRI, we aimed to identify the risk factors of PSD, develop a prediction scale with high accuracy and map key dysphagia brain areas.

**Methods:** A total of 275 acute ischemic stroke patients were enrolled in this study, and 113 (41.1%) patients were diagnosed with PSD. All patients underwent the water-swallowing test (WST) and volume-viscosity swallow test (V-VST) within first 24 h following admission to assess swallowing. Vascular factors were evaluated and MRI brain scans were obtained within 3 days after symptom onset for each participant admitted to the hospital. *T*-test, chi-squared test and Fisher’s exact test were used to investigate the associations of various patient characteristics with dysphagia, and multivariable logistic regression models were used to construct a prediction scale. Scale accuracy was assessed using receiver operating characteristic (ROC) analysis. We extracted white matter hyperintensities for each patient as potential brain lesions. Voxel-based lesion-symptom mapping (VLSM) was used to identify key brain areas for dysphagia.

**Results:** Risk factors related with PSD were older age, history of atrial fibrillation, higher fasting blood glucose, NIH stroke scale, TOAST classification, progressive stroke, middle cerebral artery lesion and anterior cerebral artery lesion. Three variables with most significant associations, including NIH stroke scale, TOAST classification and progressive stroke, combined with age and gender, were used to construct a dysphagia prediction scale with high accuracy (AUC = 0.86). VLSM identified left inferior parietal gyrus as a key brain region for PSD.

**Conclusion:** Risk factors of PSD were identified and a predictive model of dysphagia was constructed intelligently and automatically. The left inferior parietal gyrus was identified as a key brain area for dysphagia, which provides a new symptom-based treatment target for early rehabilitation in the future.

## Introduction

Non-invasive magnetic resonance imaging (MRI) provides high sensitivity and specificity for ischemic stroke. The latest developments of various MRI imaging techniques provide a comprehensive understanding of brain function. It was widely recognized that MRI play critical role in the diagnosis of stroke, particularly in the hyperacute stage. The applications of MRI make sense to identify AIS patients rapidly and facilitate clinical decision making to convert severe stroke to minor stroke.

Swallowing is a complex and bilateral neuromuscular mechanism that requires multiple brain regions to control the involved muscles and structures (Leopold and Daniels, 2010). Post-stroke dysphagia (PSD) is a common and disabling condition that occurs in up to 70% of patients, and it is associated with pneumonia, malnutrition, prolonged hospital stay, increased mortality and poor long-term outcome. Mild and moderate dysphagia often resolve within the first week, but almost one-third of patients develop post-stroke pneumonia that requires treatment (Hinchey et al., 2005).

Therefore, early recognition and management may reduce the incidence of complications, but these goals remain challenging in clinical practice. PSD remains a neglected area of research despite its high impact on prognosis. Several studies identified the prevalence of PSD and risk factors to predict dysphagia. Elderly patients with stroke have a greater chance to present swallowing dysfunction because of a reduced cough reflex, alterations in swallowing, and breathing coordination (Marik and Kaplan, 2003). The National Institute of Health Stroke Scale (NIHSS) score may be used as an adjunct to predict PSD with moderate sensitivity and specificity (Labeit et al., 2018).

Previous research showed associations between distinct lesion locations and PSD. The brain stem (especially the medulla and pons) is the central control of swallowing, but cortical and subcortical regions play integral roles in mediating swallowing (Wilmskoetter et al., 2019). An independent correlation between leukoaraiosis severity and PSD was previously reported, which emphasizes the important role of subcortical white matter in swallowing. Disruption of cortical–subcortical white matter connections resulted in dysphagia by lowering the threshold of the input to the swallowing center (Toscano et al., 2015). Previous reports stated that brain lesions in the right hemisphere caused pharyngeal dysphagia, and lesions in the left hemisphere caused dysfunction during the oral phase of swallowing (Ickenstein et al., 2005). However, there are no definite conclusions about whether one hemisphere is more involved than the brain stem and whether one hemisphere was dominant.

All of these findings are inconsistent due to the comparatively small sample sizes, different dysphagia screening methods, various inclusion criteria and inaccurate classification of brain regions. Most previous research lacked comprehensive clinical and imaging studies. Therefore, it is urgent to find a clear picture of PSD with risk factors and clinical outcomes to improve stroke healthcare settings. The present study took advantage of comprehensive clinical data from early clinical swallowing examinations and voxel-based image analysis approaches. The aims of this study were (1) to establish risk factors and construct a predictive model of dysphagia and (2) map lesion-symptoms for PSD to predict PSD in AIS and help healthcare settings.

## Materials and Methods

### Study Design and Participants

Study participants were from acute ischemic stroke (AIS) patients admitted to our stroke unit between October 2017 and May 2018. Inclusion criteria were based on the diagnosis of AIS as confirmed on diffusion-weighted MRI (DWI). Patients with the following conditions were excluded from the study: (1) preexisting dysphagia; (2) concomitant intracerebral hemorrhage; (3) brain tumors; (4) severe hepatic and renal dysfunction; (5) end-stage severe disease; and (6) concomitant diseases likely to cause dysphagia, including dementia. All subjects were divided into two groups according to the following swallowing assessment: (1) patients with PSD; and (2) patients without PSD.

PSD was assessed using the water-swallowing test (WST) and volume-viscosity swallow test (V-VST). WST was performed using 30 ml of water while sitting at a 90° angle (Osawa et al., 2013). V-VST is a clinical assessment that uses boluses of different volumes (5, 10, and 20 ml) and viscosities (thin liquid, nectar-like, and spoon thick) in combination with a pulse-oximeter to evaluate the efficacy and safety of swallowing with minimum risk for the patient. Signs and symptoms of impaired efficacy of swallowing included oral residue, efficiency of labial seal, fractional swallow and pharyngeal residue. Signs of impaired safety of swallowing included changes in voice quality (including wet voice), coughing and decreased oxygen saturation (SpO2) ≥ 3% from basal level (Rofes et al., 2018). Validation studies showed excellent psychometric properties for V-VST (sensitivity 0.94 and specificity 0.88) in detecting PSD (Vilardell et al., 2017). V-VST was performed within 24 h after admission before oral feeding, with the exception of patients with low levels of consciousness. Patients who presented with any signs of impaired efficacy and/or safe swallowing were considered as having PSD. A trained neurologist who was blinded to the patient’s clinical results examined all patients.

The ethics committee of our hospital approved the study protocol (No. 2021041). Informed consent was obtained from all patients or legal guardians if the patient’s communication and/or understanding were impaired.

### Imaging Procedures

All patients were scanned in a 3T MR scanner (MAGNETOM Skyra; Siemens Healthineers, Erlangen, Germany). A 20-channel brain array coil was used for signal reception. The images obtained included transverse T1-weighted turbo spin-echo (TSE) images [repetition time (ms)/echo time (ms), 700/14; section thickness, 3 mm; intersection gap, 0.5 mm; field of view, 25 cm; matrix, 384 × 336] and transverse T2-weighted TSE images [repetition time (ms)/effective echo time (ms) 6,000/124; section thickness, 3 mm; intersection gap, 0.3 mm; field of view, 25 cm; matrix, 384 × 336]. DWI was obtained to calculate an apparent diffusion coefficient using a 2D echo planar imaging sequence with multiple *b*-value acquisitions (0, 100, 800, 1,000, and 1,500 s/mm^2^), with the diffusion-sensitizing gradients applied along the X, Y, and Z axes. MRI brain scans were obtained within 3 days after symptom onset for each participant admitted to the hospital. Lesions were localized according to hemisphere (right, left, or bilateral).

The image preprocessing includes registration for aligning all images into the same coordinate system, imaging normalization and white matter hyperactivity extraction. Although the images were acquired during the same session, a certain amount of subject motion and movement is unavoidable between the sequences, which leads to image misalignment. Basically, we extracted white matter hyperintensities from T2 images and stroke lesions from DWI images in the similar way with a stricter parameter, 0.5. In addition, T1 images ware applied to help image preprocessing including normalizations. For each subject, the T2 images were aligned with the T1 images using the 3D rigid body image registration algorithm proposed by Ashburner et al. (2000). We used the non-rigid normalization toolbox and transformed the T1 images into a standard template of MNI (Montreal Neurological Institute) space (Smith et al., 2018). T2 and DWI images were also transformed into standard space based on the deformation field of the corresponding T1 images. We used the Lesion Segmentation Toolbox (Schmidt et al., 2012) to identify white matter hyperintensities (lesion localized) and stroke lesions automatically of white matter regions and white matter anatomy of gray matter regions for each subject. We subtracted stroke lesions found by DWI images from white matter hyperintensities extracted by T2 images, which were considered potential lesions (Debette and Markus, 2010). Two trained neurologists who were blinded examined all of the preprocessing results to assure the imaging quality.

### Vascular Risk Factors and Other Co-variates

We collected the following variables: age, gender, medical history (including hypertension, diabetes mellitus, smoking, history of stroke, atrial fibrillation, and other heart diseases), clinical data on admission (including relevant laboratory indicators, stroke severity as measured by the NIHSS, and thrombolytic treatment), location of stroke (left, right, bilateral hemispheres or posterior circulation) and affected vessels [anterior cerebral artery (ACA), middle cerebral artery (MCA), internal carotid artery (ICA) and posterior cerebral artery (PCA)]. The etiology of stroke was determined according to TOAST, which refers to five classifications: (1) large-artery atherosclerosis (LAA), (2) cardioembolism (CE), (3) small-vessel occlusion (SVO), (4) stroke of other determined etiology (SOE), and (5) stroke of undetermined etiology (SUE). The treating team made the diagnoses of progressive stroke. The following diagnostic criteria were used for progressive stroke: (a) the disease course extended from 6 h to 7 days; (b) the primary nervous symptoms and signs of the cerebral infarction progressively worsened after regular treatment for cerebral infarction, and the NIHSS score increased no less than 2 points (Zhang et al., 2017).

### Statistical Tests

Continuous variables are presented as the means ± standard deviation (SD) or minimum-maximum and were analyzed using Student’s *t*-test and one-way ANOVA. Categorical data were examined using the chi-squared test or Fisher’s exact test. A multivariate logistic regression was used to construct a PSD prediction scale, and its accuracy was assessed using receiver operating characteristic (ROC) analysis with *p*-values extracted by permutation tests. Voxel-based lesion-symptom mapping (VLSM) (Bates et al., 2003) was used to analyze the relationship between tissue lesions (white matter hyperintensities) and PSD on a voxel-by-voxel basis to help identify key brain areas of PSD. For each voxel, patients were divided into two groups according to whether they did or did not have a lesion affecting that voxel. PSD or not were then compared for these two groups, yielding a logistic regression for each voxel. Moreover, we added covariates (e.g., age, gender, and lesion size) in the regression analysis to remove their effects. In addition, we applied cluster analysis to do multiple comparison corrections. Statistical analyses were performed using MATLAB software, version 2019b (MathWorks Inc.). A statistical threshold corrected at cluster-level for familywise error correction at *p* = 0.05 was set to reduce type-1 errors in the voxel-level analysis.

## Results

**FIGURE 1:**
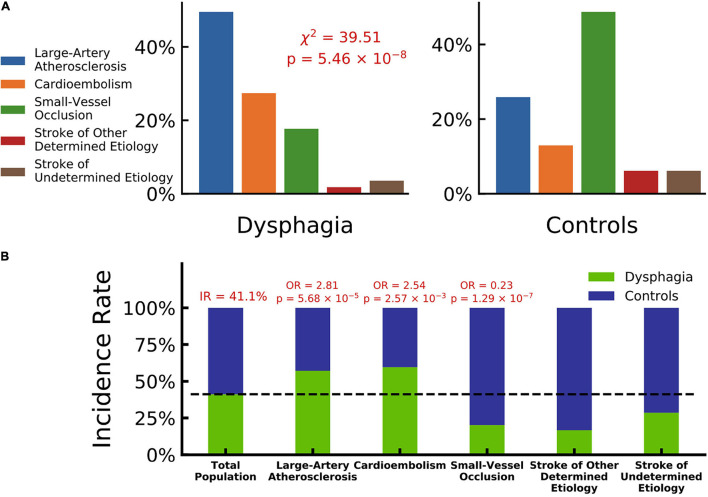
Dysphagia and stroke associations by TOAST classification. **(A)** Composition ratios of TOAST classification (blue: large-artery atherosclerosis, orange: cardioembolism, green: small-vessel occlusion, violet: stroke of other determined etiology and red: stroke of undetermined etiology) in patients with dysphagia and controls, which indicates a significantly strong association between dysphagia and stroke by TOAST classifications (Chi-squared test, χ^2^ = 39.51, *p* = 5.46 × 10^–8^). **(B)** Incidence rate of dysphagia in different TOAST classifications (patients with dysphagia in green and controls in blue). The incidence rate of total population is 41.1%. Large-artery atherosclerosis and cardioembolism hold odd ratios significantly larger than 1 with dysphagia (OR = 2.81, *p* = 5.68 × 10^–5^ and OR = 2.54, *p* = 2.57 × 10^–3^), while small-vessel occlusion holds an odd ratio significantly lower than 1 (OR = 0.23, *p* = 1.29 × 10^–7^).

A total of 275 patients fulfilled the inclusion criteria and were included in the study (Supplementary Figure 1 summarized details of study recruitment). A total of 113 (41.1%) of the patients had PSD: 44 patients were assessed as V grade in the WST and were directly given nasal feeding. Other 69 patients were assessed using the V-VST. A total of 14.5% (*n* = 10) had clinical signs of impaired safety, 18.8% (*n* = 13) had impaired efficacy, and 66.7% (*n* = 46) had both swallowing impairments. The demographic and clinical characteristics of the study population and the differences between PSD and non-PSD patients are shown in Table 1. The groups significantly differed in age, and PSD patients were older than the non-PSD patients (67.92 ± 12.22 vs. 63.38 ± 13.19 years, *t* = 2.89, *P* < 0.01). Patients with history of AF (29.9% vs. 9.4%, χ^2^ = 11.05, *P* < 0.01) and higher FBG (6.59 ± 2.84 vs. 5.54 ± 1.74 μmol/l, *t* = 3.78, *P* < 0.01) were more likely to suffer from dysphagia. PSD patients showed higher scores in NIHSS (10.50 ± 6.95 vs. 3.38 ± 3.30, *t* > 11.34, *P* < 0.01) and often were characterized by concomitant stenosis or occlusion of the MCA (56.9% vs. 21.8%, χ^2^ = 11.85, *P* < 0.01) and ACA (3.7% vs. 1.2%, Z = 7.05, *P* < 0.01). Patients with progressive stroke had a higher chance of exhibiting PSD (31.4% vs. 3.2%, χ^2^ = 28.03, *P* < 0.01). We did a ROI analysis of stroke lesions in PSD and identified temporal lobe (815.40 ± 1305.77 vs. 548.93 ± 723.34, *t* = 2.02, *P* < 0.05), hippocampus (318.61 ± 331.74 vs. 236.38 ± 273.14, *t* = 2.06, *P* < 0.05), basal nucleus (717.36 ± 701.56 vs. 528.68 ± 495.54, *t* = 2.42, *P* < 0.01) and internal capsule (303.10 ± 584.71 vs. 118.25 ± 308.46, *t* = 3.18, *P* < 0.001) in left hemisphere to be associated with PSD as shown in Table 1. Post-stroke pneumonia was not associated with a higher chance of having PSD. A strong association was found between dysphagia and stroke using TOAST classifications (χ^2^ = 39.51, *P* < 0.01). We investigated the associations of dysphagia and stroke using the TOAST classification as shown in Figure 1. Patients with LAA and CE suffered dysphagia more often (LAA: OR = 2.81, *P* < 0.01; CE: OR = 2.54, *P* < 0.05), and patients with SVO suffered less often (OR = 0.23, *P* < 0.01). Patients of other determined and UD did not show significant differences.

**TABLE 1:** Demographic and clinical data of patients with dysphagia and controls.

Demographic and clinical data	Dysphagia (*n* = 113)	Controls (*n* = 162)	t/Z/χ ^2^	p
Age (years)	67.92 ± 12.22, 31.00–93.00	63.38 ± 13.19, 22.00–85.00	*t* = 2.89	2.10 × 10^–3*^
Gender male/female	75/38	107/55	χ^2^ = 0.00	0.96
Systolic blood pressure (mmHg)	145.37 ± 21.33, 103.00–201.00	142.77 ± 21.01, 104.00–207.00	*t* = 1.00	0.32
Diastolic blood pressure (mmHg)	81.30 ± 13.12, 40.00–118.00	80.62 ± 12.89, 7.00–117.00	*t* = 0.43	0.67
History of hypertension yes/no	90/23	111/51	χ^2^ = 4.19	0.04
History of diabetes yes/no	26/87	31/131	χ^2^ = 0.61	0.44
Smoking yes/quit/no	30/4/79	39/4/119	*Z* = 0.32	0.75
History of AF^†^ yes/no	26/87	14/148	χ^2^ = 11.05	8.85 × 10^–4*^
Other heart diseases yes/no	6/107	5/157	*Z* = 0.90	0.37
Previous stroke yes/no	23/90	21/141	χ^2^ = 2.71	0.10
Triglyceride (mmol/L)	1.27 ± 0.53, 0.51–3.83	1.47 ± 0.95, 0.38–9.11	*t* = −1.97	0.05
Total cholesterol (mmol/L)	4.26 ± 0.99, 2.21–6.93	4.19 ± 1.09, 0.65–8.21	*t* = 0.55	0.58
LDLC (mmol/L)	2.43 ± 0.81, 0.69–4.88	2.38 ± 0.83, 0.65–5.15	*t* = 0.56	0.57
Creatinine (μmol/L)	70.19 ± 20.08, 38.50–191.70	69.37 ± 17.12, 32.50–121.00	*t* = 0.36	0.72
Uric acid (μmol/L)	285.82 ± 95.45, 9.30–553.50	297.65 ± 88.86, 92.50–574.20	*t* = −1.05	0.29
Fasting blood glucose (μmol/L)	6.59 ± 2.84, 3.43–19.03	5.54 ± 1.74, 3.34–12.67	*t* = 3.78	1.00 × 10^–4*^
Homocysteine^‡^ (μmol/L)	12.90 ± 10.74, 2.40–97.00	11.43 ± 7.82, 3.10–56.20	*t* = 1.17	0.12
Hemoglobin A1c^‡^ (%)	6.93 ± 1.88, 3.70–12.30	6.70 ± 1.88, 4.90–14.80	*t* = 0.80	0.21
ECG^†^ (AF) yes/no	30/83	19/143	χ^2^ = 9.98	1.58 × 10^–3*^
NIH stroke scale	10.50 ± 6.95, 0.00–36.00	3.38 ± 3.30, 0.00–19.00	*t* = 11.34	5.50 × 10^–25**^
NIH stroke scale (After discharge)	8.58 ± 5.98, 0.00–35.00	2.22 ± 2.55, 0.00–16.00	*t* = 12.06	1.93 × 10^–27**^
TOAST classification†	56/31/20/2/4	42/21/79/10/10	χ^2^ = 39.51	5.46 × 10^–8**^
Progressive stroke yes/no	27/86	5/157	χ^2^ = 28.03	1.20 × 10^–7**^
Lesioned hemi left/right/both	43/52/18	84/55/23	χ^2^ = 5.37	0.07
Brain stem lesioned yes/no	25/88	25/137	χ^2^ = 2.00	0.16
MCA^†^ Affected yes/no	41/72	29/133	χ^2^ = 11.85	5.75 × 10^–4*^
PCA^†^ Affected yes/no	5/108	16/146	χ^2^ = 2.81	0.09
ICA^†^ Affected yes/no	21/92	16/146	χ^2^ = 4.33	0.04
ACA^†^ Affected yes/no	4/109	2/160	*Z* = 7.05	1.76 × 10^–12**^
BA^†^ Affected yes/no	6/107	4/158	*Z* = 0.00	1.00
VA^†^ Affected yes/no	5/108	3/159	*Z* = 0.00	1.00
Thrombolytic yes/no	19/94	23/139	χ^2^ = 0.35	0.55
Epilepsy yes/no	3/110	4/158	*Z* = 0.00	1.00
Lesion size of temporal lobe (left) (mm^∧^3)	815.40 ± 1305.77	548.93 ± 723.34	*t* = 2.02	0.02
Lesion size of hippocampus (left) (mm^∧^3)	318.61 ± 331.74	236.38 ± 273.14	*t* = 2.06	0.02
Lesion size of basal nucleus (left) (mm^∧^3)	717.36 ± 701.56	528.68 ± 495.54	*t* = 2.42	8.20 × 10^–3^
Lesion size of internal capsule (left) (mm^∧^3)	303.10 ± 584.71	118.25 ± 308.46	*t* = 3.18	8.00 × 10^–4*^

*Continuous data are shown as mean ± SD, minimum and maximum values in patients with dysphagia and controls with statistical significance based on two sample t-test. Categorical data differences in patients and controls are represented with statistical significance based on chi-squared test (χ^2^ and p) or Fisher exact test (Z and p). *p < 0.005, **p < 1.00 × 10^–6^.

*^†^AF, refers to atrial fibrillation; LDLC, refers to low density lipoprotein cholesterol; ECG, refers to Electrocardiogram; MCA, refers to middle cerebral artery; PCA, refers to posterior cerebral artery; ICA, refers to internal carotid artery; ACA, refers to anterior cerebral artery; BA, refers to basilar artery and VA, refers to vertebral artery. TOAST refers to five classifications: (1) large-artery atherosclerosis, (2) cardioembolism, (3) small-vessel occlusion, (4) stroke of other determined etiology, and (5) stroke of undetermined etiology.*

*^‡^87 patients with dysphagia and 131 controls took part in Homocysteine tests, while 66 patients with dysphagia and 100 controls attended Hemoglobin A1c tests.*

### Post-stroke Dysphagia Risk Factors and Prediction Scale

In order to avoid the influence of multiple comparison, we raised the threshold to (*p* < 1.00 × 10^–6^ with ** in Table 1) when doing multivariate analysis. The three variables with the most significant associations, NIH stroke scale, TOAST classification and progressive stroke, were combined with age and gender and used to construct a dysphagia prediction scale using a multivariate logistic model. As shown in Table 2, NIH stroke scale (OR = 1.27, *t* = 6.30, *P* < 0.01) and progressive stroke (OR = 5.65, *t* = 2.97, *P* < 0.01) showed the most significant associations with PSD in multivariate statistical analysis, and the other variables, including age, gender and TOAST classifications, showed no association.

**TABLE 2:** Multivariable logistic regression model for predicting patients with dysphagia.

Variables	Odds ratio	95% CI	t	*P-*value
Age	1.03	1.00, 1.06	1.72	0.09
Gender	1.78	0.87, 3.66	1.59	0.11
NIH stroke scale	1.27	1.18, 1.38	6.30	3.04 × 10^–10^**
Large-artery atherosclerosis	1.36	0.39, 4.79	0.48	0.63
Cardioembolism	0.88	0.20, 3.90	–0.17	0.86
Small-vessel occlusion	0.59	0.15, 2.23	–0.79	0.43
Progressive stroke	5.65	1.80, 17.76	2.97	0.0029*

*p < 0.005, **p < 1.00 × 10^–^^6^.

**FIGURE 2:**
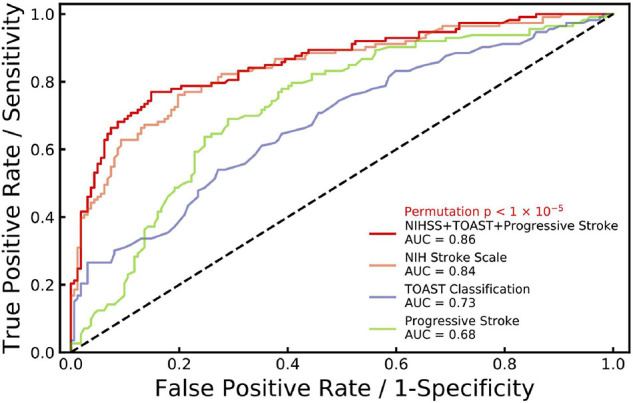
ROC curves generated for stroke with dysphagia. Receiver operating characteristic (ROC) curve is generated for NIH stroke scale (NIHSS), TOAST classification and progressive stroke (age and gender effects included) with dysphagia (red line, AUC = 0.86, permutation *p* < 1 × 10^–5^) based on multiple logistic regression. In addition, ROC curves are generated separately for NIHSS (orange, AUC = 0.84), TOAST classification (blue, AUC = 0.73) and progressive stroke (green, AUC = 0.68) with age and gender effects included.

A ROC analysis was performed to examine the accuracy of the dysphagia prediction scale. As shown in Figure 2, NIH stroke scale, TOAST classification and progressive stroke together showed a significantly high AUC (area under the ROC curve) of 0.86 with a permutation *P* < 1 × 10^–5^, which indicated higher accuracy of the dysphagia prediction scale. The AUC of predictions based on NIH stroke scale (orange curve, AUC = 0.84), TOAST classification (blue curve, AUC = 0.73) and progressive stroke (green curve, AUC = 0.68) separately are also represented. Moreover, in the samples with images, as shown in Supplementary Figure 3, NIH stroke scale, TOAST classification and progressive stroke together showed AUC of 0.84 with a permutation *P* < 1 × 10^–5^. NIH stroke scale, TOAST classification, progressive stroke and regional lesion location together showed a significantly higher AUC of 0.85. Age and gender effects were included in the multivariate logistic model to construct prediction scales.

Once the predictive variables were determined and the accuracy was confirmed by the ROC analysis, an online calculator tool was constructed to help neurologists to acquire PSD risks of AIS patients intelligently and automatically (with risk scores and classifications included, http://180.167.250.222:10080/Online-Dysphagia-Risk-Calculator-Tool-for-AISPatients.html, Supplementary Figure 2).

### Voxel-Based Lesion-Symptom Mapping of Post-stroke Dysphagia

**FIGURE 3:**
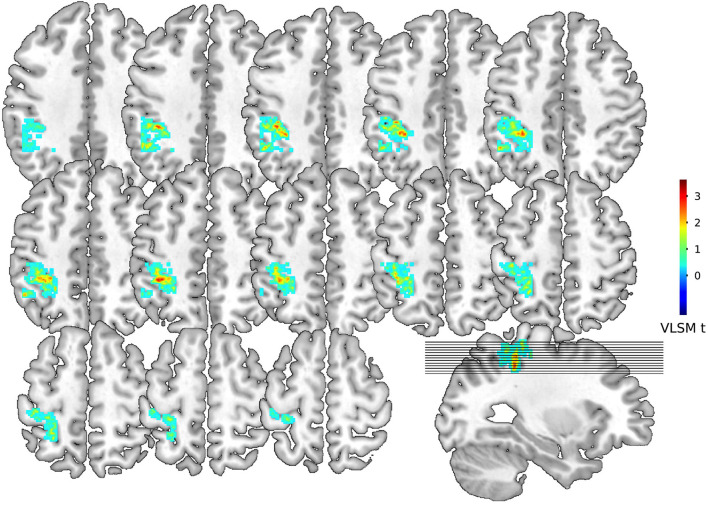
Voxel-based lesion-symptom mapping for dysphagia symptoms. Representative slices from VLSM maps computed for dysphagia symptoms of 275 patients with stroke. These maps are colorized depictions of logistic regression analysis results evaluating patient performance on a voxel-by-voxel basis. The warmer the color is, patients with lesions on the corresponding voxel hold higher chance to develop dysphagia. Age, gender, NIH stroke scale, TOAST classification and progressive stroke effects have been removed.

The VLSM was used to identify lesions (white matter hyperintensities) that were strongly connected with PSD in 275 patients with AIS. We found a list of voxels with potential relationships. To eliminate noise effects, we performed a cluster analysis and found that the largest cluster (greater than 100 mm^3^) fell on the left IPG with the center at MNI coordinates: X = −38, Y = −39, Z = 51, *t* = 3.59, *P* < 0.0001). Figure 3 showed colorized depictions of VLSM logistic regression analysis results evaluating lesion and PSD relationships on a voxel-by-voxel basis. The left IPG had a specific and functional connection with PSD in the VLSM analysis.

## Discussion

Combining clinical symptoms with applications of MRI (T1, T2, and DWI sequences) and VLSM analysis methods, our study demonstrated two major findings in acute ischemic stroke patients: (1) potential clinical risk factors of the onset of dysphagia following acute ischemic stroke, including older age, history of atrial fibrillation, higher fasting blood glucose, NIH stroke scale, TOAST classification, progressive stroke, middle cerebral artery lesion and anterior cerebral artery lesion with a PSD prediction scale; and (2) lesions in different anatomical locations in patients with AIS showed specific and functional connections between PSD and left inferior parietal gyrus using VLSM.

MRI has been proven to be high sensitivity and specificity for ischemic stroke, particularly in the hyperacute stage. Different MRI methods have been worldwide applied in the assessment of AIS patients in clinical practice (Asaithambi et al., 2020). Combining clinical symptoms with applications of MRI, we established a predictive model of dysphagia in AIS patients intelligently and automatically. With VLSM, the left inferior parietal gyrus was identified as a key brain area for dysphagia. After the automatic operation of the conventional MRI sequences mentioned above and VLSM analysis methods, the efficiency of MRI applications was improved. The role of MRI has broadened and progressed from making diagnoses to providing important information for clinical decision making and AIS patients management. An increasing body of evidence suggest that the physiology revealed by MRI can be an advance over time in clinical decisions in the management of AIS patients, which finally can improve the prognosis of patients (Campbell and Parsons, 2018).

PSD is prevalent in hospitalized patients, and it is associated with increased mortality and comorbidities. The reported incidence ranges substantially across studies from 50 to 80% (Woodhouse et al., 2018), depending on the different evaluation methods. The prevalence of PSD (41.1%) in our study is consistent with other studies in which similar bedside clinical assessments were used.

Previous studies identified various clinical variables, such as, age, dysarthria, lesion volume and stroke severity. However, these findings were inconsistent. Our study identified certain demographic and clinical factors that influenced PSD to help identify patients with a greater risk of PSD at an early stage. Age and stroke severity are consistently considered predictors for PSD. In addition to that, a predictive model of PSD was constructed intelligently and automatically. Older age is consistently considered as a risk factor for dysphagia after stroke. Older patients are particularly vulnerable to PSD because of multiple age-related changes. On the one hand, elderly patients cannot compensate for sarcopenia (Wirth et al., 2016). On the other hand, a broader cortical activation in both hemispheres was identified measured by MEG during swallowing in elderly subjects compared to the young (Teismann et al., 2010). As a consequence, it is assumed that cortical lesions in elderly patients are more likely to lead to PSD due to an already reduced compensatory reserve. With regards to gender, the impact on PSD has not yet been systematically explored. One retrospective study identified that female sex was associated with prolonged dysphagia and increased death (Smithard et al., 2007). Considerable research found that stroke severity, as measured by NIHSS score, was an independent risk factor for PSD adjusting for other variables (Labeit et al., 2018; Toscano et al., 2019). Two studies evaluated the correlation of NIHSS score and dysphagia severity. And one article found that a cutoff value of 10 points carried the best sensitivity for predicting PSD (Jeyaseelan et al., 2015). Another article set the cutoff value at 12 points (Okubo et al., 2012). However, no research focused on the relationship between stroke subtype based on the TOAST criteria and PSD. The only related article found no significant difference between TOAST classification and the requirement for percutaneous endoscopic gastrostomy tube insertion in patients after stroke (Li et al., 2014). Our study provided evidence that patients with LAA and CE were more likely to suffer from PSD. In general, patients with LAA and CE had a higher NIHSS score than patients with other subtypes (Adams et al., 1999). Therefore, one potential explanation is that patients with LAA and CE tend to have severe neurological deficits (Tan et al., 2018). There was little literature on the relationship between progressive stroke and PSD. The mechanism of progressive stroke was not clear. The hypotheses involved included propagation of thrombus, development of brain edema, production of inflammatory cytokines and metabolic factors. Besides, progressive stroke was reported to be positively correlated with large vessel disease or branch atheromatous disease (Saia and Pantoni, 2009). Our study confirmed that an increased NIH stroke scale and progressive stroke were associated with PSD in patients with AIS. The PSD risk increased 1.27 times with every one NIHSS score increase, and patients with progressive stroke had a 5.65-times higher PSD risk. Higher NIHSS stroke scale and progressive stroke should be considered two main symptom targets during healthcare settings for PSD.

A strong dominance of left hemispheric lesions was observed in our study, and this result confirmed previous research that unilateral hemispheric lesions produce PSD (Daniels et al., 1996). However, there was no unified conclusion about the dominant role of the left and right cerebral hemispheres. Some functional imaging showed time-dependent shifts of neural activation from the left to right sensorimotor cortex during swallowing (Teismann et al., 2009). Recent evidence suggested that swallowing was represented bilaterally, but asymmetrically, with interindividual and characteristic differences (Saito et al., 2016).

Previous studies attempted to identify the association between the location of brain lesions and PSD based on functional neuroimaging. The nucleus ambiguus in the rostral and ventrolateral medulla, the nucleus tractus solitarius and the reticular formation contain the central pattern generator for swallowing (Daniels et al., 2017). Cortical regions associated with primary and secondary sensorimotor areas were identified as important in swallowing in neuroimaging studies. Previous studies showed several regions related to swallowing, such as supramarginal gyrus, the precentral gyrus, post-central gyrus, insula, and cingulate cortex (Rolls, 2016). Multiple subcortical and white matter regions were also involved in abnormal swallowing (Malandraki et al., 2009). Gonzalez-Fernandez et al. (2008) found a positive association between dysphagia and acute stroke involving the internal capsule after adjusting for stroke severity (NIHSS) and stroke volume. Daniels and Foundas (1999) identified that periventricular white matter was highly associated with dysphagia. These regions may provide interconnections between cortical swallowing centers and the central pattern generator. There is no doubt that brain stem is the central control of swallowing and plays an absolutely important role in PSD as evidenced in by the high incidence of PSD in cases with lateral medullary stroke. However, in our study, only 50/275 (18.2%) of the patients were with brain stem lesions. The absence of significant effects in brain stem lesions in our study may result from small patient collectives.

There was no consistent conclusion about the relationship between brain lesions and the occurrence of PSD. A number of studies in the past have strived to identify lesion patterns that predict dysphagia (Galovic et al., 2013; Suntrup-Krueger et al., 2017). However, all of these findings were inconsistent due to a lot of reasons. First, the comparatively small sample sizes had limited our ability to predict which patients were more likely to develop swallowing dysfunction. Moreover, dysphagia is time-dependent and conclusions about the association between stroke locations and dysphagia required early assessment of both stroke and swallowing. Not only that, but studies were often confined to a low number of roughly classified brain regions of interest (Suntrup et al., 2015). The limitations of functional imaging may also account for this lack of consensus. Functional neuroimaging requires a long time, and patients inevitably move, which compromises the quality of imaging data. There are also different results from different neuroimaging methods. Imaging findings change over time due to treatment, disease duration and severity (Fox, 2018).

The present study showed that lesions in different anatomical locations presented strong connections between the left IPG and PSD using VLSM. To the best of our knowledge, the IPG is widely accepted as associated with the egocentric, perceptual, exploratory extrapersonal, and personal, bodily components of neglect (Vallar and Calzolari, 2018). The roles of the IPG in conceptual processing and context-driven word production were also observed previously (Piai et al., 2018). The most common site of involvement in this study was the IPG. Swallowing is a complicated and multisensory process that depends on several sensory modalities. Therefore, it was not surprising that the IPG, as the secondary somatosensory cortex, may contribute to sensorimotor integration during swallowing. Our findings suggest the importance of intact sensory afferents for the motor output network of swallowing (Galovic et al., 2013). This new approach using network mapping identified previously unappreciated connections in patients with PSD and provide a new symptom-based treatment target. The current treatments for PSD are diet improvement, and compensatory and rehabilitation methods. However, these rehabilitation methods are primarily experience-based for dysfunction of a typical swallowing organ, and no evidence shows that these methods may be used as a routine clinical treatment for dysphagia after stroke (Mitchell, 2016). Transcranial magnetic stimulation (TMS) is a tool to alter brain activity in CNS disease and has been used successfully to study swallowing. It was reported that both ipsilesional high frequency and contralesional low frequency TMS could improvement in the symptoms and signs of dysphagia in the acute and chronic stroke (Michou and Hamdy, 2009). Lin et al. (2018) indicated that vagus nerve modulation using TMS in patients with stroke involving the brainstem can facilitate the recovery of swallowing function. In more recent literature, TMS was approved to have a positive value on the treatment of dysphagia after stroke by several authors (Verin and Leroi, 2009; Khedr and Abo-Elfetoh, 2010). Therefore, the IPG may be a new symptom-based treatment target of PSD. Therefore, the IPG may be a new symptom-based treatment target of PSD.

Our study had some limitations. First, we must consider that PSD in our study was not confirmed via instrumental testing, such as videofluoroscopy. Instead, swallowing was assessed clinically using bedside evaluations (WST and VVST), which may lack the ability to evaluate silent aspiration. Furthermore, instrumental validation could have allowed for a more detailed score to evaluate severity of PSD. However, instrumental assessment of swallowing function existed limitation in clinical practice, especially to AIS patients. The bedside evaluations we performed were not only quite simple and easy, but also had good sensitivity and specificity, which was confirmed as a formalized assessment for dysphagia by both the European Society for Swallowing Disorders (ESSD) and expert group on PSD and nutritional management in China (Rofes et al., 2013). Second, the severity of PSD should be further assessed to examine the factors that affect the severity of dysphagia. Third, No FLAIR images were acquired which would be ideal for our study. FLAIR can more clearly show the lesions that are concealed by the high signal of cerebrospinal fluid on conventional T2WI, especially in the periventricular, brain surface, and subarachnoid area, which improves the sensitivity of diagnosis. We will pay particular attention to this point in future research.

## Conclusion

In conclusion, the present study showed that dysphagia was a frequent symptom in patients with AIS. Possible predictors of PSD were identified and included older age, history of atrial fibrillation, higher fasting blood glucose, NIH stroke scale, TOAST classification, progressive stroke, middle cerebral artery lesion and anterior cerebral artery lesion, which were used to construct a dysphagia prediction scale with high accuracy. An online predictive model of dysphagia was constructed to help neurologists to acquire PSD risks of AIS patients intelligently and automatically. The left inferior parietal gyrus was a key brain region of PSD associated with dysphagia after AIS and may be a new symptom-based treatment target.

## Data Availability Statement

The raw data supporting the conclusions of this article will be made available by the authors, without undue reservation.

## Ethics Statement

The studies involving human participants were reviewed and approved by the Ethics Committee of the First Affiliated Hospital of Soochow University approved the study protocol (No. 2021041). The patients/participants provided their written informed consent to participate in this study. Written informed consent was obtained from the individual(s) for the publication of any potentially identifiable images or data included in this article.

## Author Contributions

LZ and XT conceived the research and wrote the main manuscript text. CW and DD participated in the recruitment of the sample population. JZ, YZ, and SD acquired the data and analyzed the results. YK and XC helped in interpreted the results and revised the manuscript. CL designed the online calculator tool to acquire PSD risks of AIS patients. YY and QF guided the process, interpreted the results, and revised the manuscript. All authors read and approved the manuscript.

## Conflict of Interest

CL was employed by company Shanghai Zhiyu Software Technology Co., Ltd. The remaining authors declare that the research was conducted in the absence of any commercial or financial relationships that could be construed as a potential conflict of interest.

## Publisher’s Note

All claims expressed in this article are solely those of the authors and do not necessarily represent those of their affiliated organizations, or those of the publisher, the editors and the reviewers. Any product that may be evaluated in this article, or claim that may be made by its manufacturer, is not guaranteed or endorsed by the publisher.

## Supplementary Material

Supplementary Figure 1 Details of study recruitment. AIS, acute ischemic stroke; TIA, transient ischemic attack.

Supplementary Figure 2 Online dysphagia risk calculator tool for patients with acute ischemic stroke. The tool is available at http://180.167.250.222:10080/Online-Dysphagia-Risk-Calculator-Tool-for-AISPatients.html.

Supplementary Figure 3 ROC curves generated for stroke with dysphagia. Receiver operating characteristic (ROC) curve is generated for NIH stroke scale (NIHSS), TOAST classification, progressive stroke and regional lesion location (age and gender effects included) with dysphagia (red line, AUC = 0.85, permutation *p* < 1 × 10^–5^) based on multiple logistic regression. ROC curves are generated for NIHSS, TOAST classification and progressive stroke (blue, AUC = 0.84) with age and gender effects included.

## Abbreviations

PSD: post-stroke dysphagia
VLSM: voxel-based lesion-symptom mapping
IPG: inferior parietal gyrus
NIHSS: National Institutes of Health Stroke Scale
WST: water-swallowing test
V-VST: volume-viscosity swallow test.
