# Supplementary figures and images for: Predictive Model of Dysphagia and Brain Lesion-Symptom Mapping in Acute Ischemic Stroke
Source: Front Aging Neurosci. 2021 Oct 20;13:753364. doi: 10.3389/fnagi.2021.753364 (PMC8564389; doi:10.3389/fnagi.2021.753364)

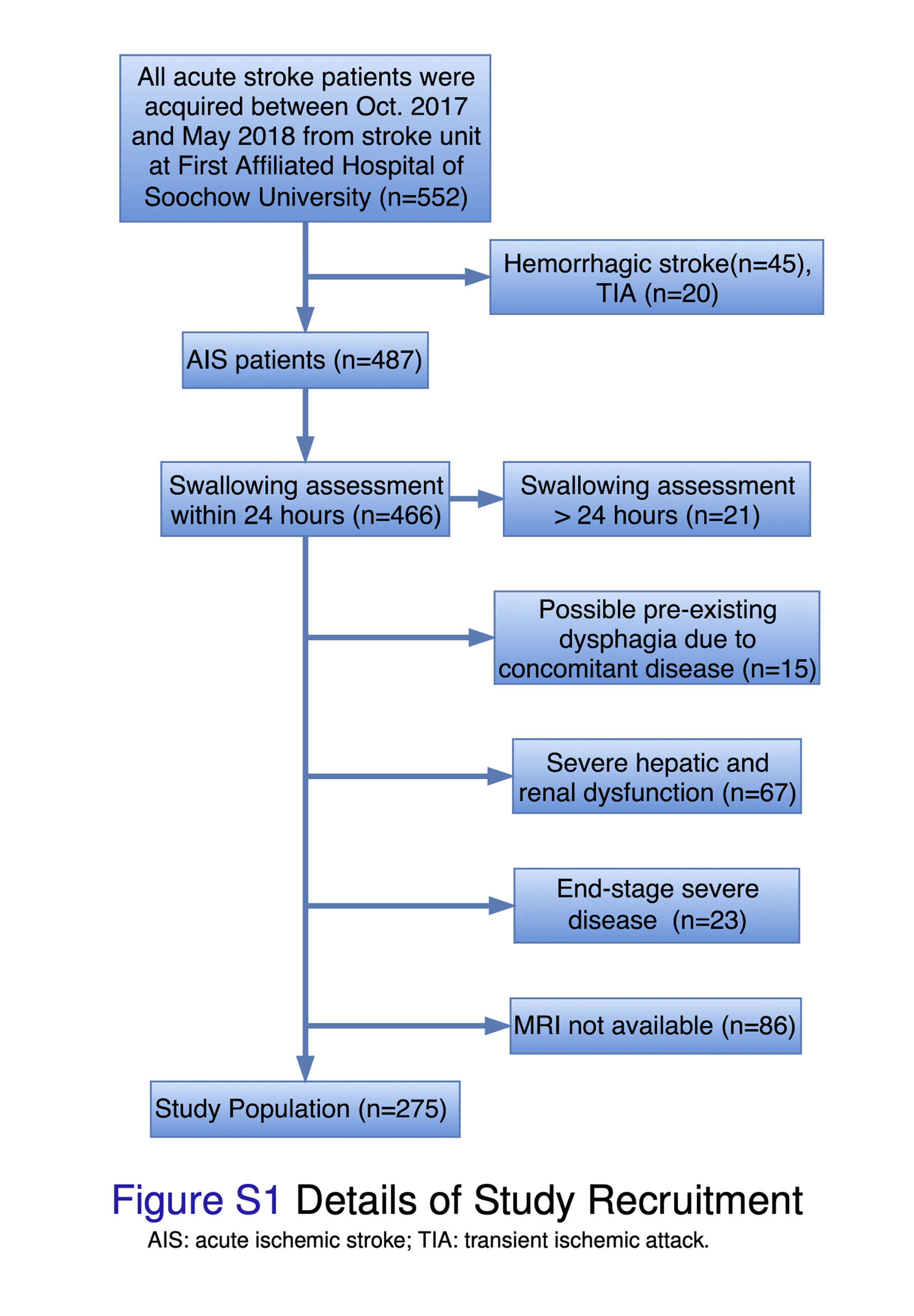

Supplement: Supplementary Figure 1 — Details of study recruitment. AIS, acute ischemic stroke; TIA, transient ischemic attack. [file Image_1.tif]

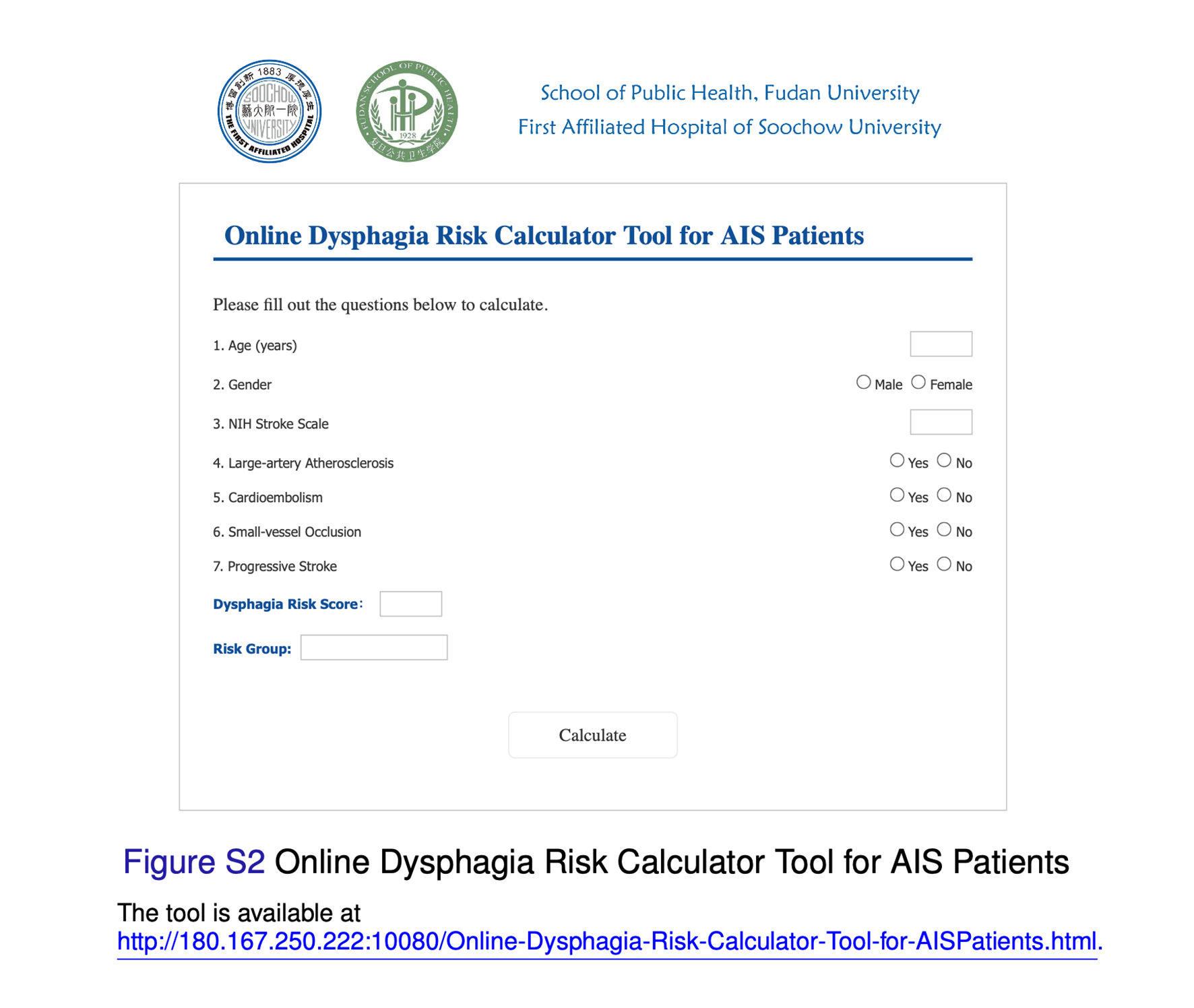

Supplement: Supplementary Figure 2 — Online dysphagia risk calculator tool for patients with acute ischemic stroke. The tool is available at http://180.167.250.222:10080/Online-Dysphagia-Risk-Calculator-Tool-for-AISPatients.html. [file Image_2.tif]

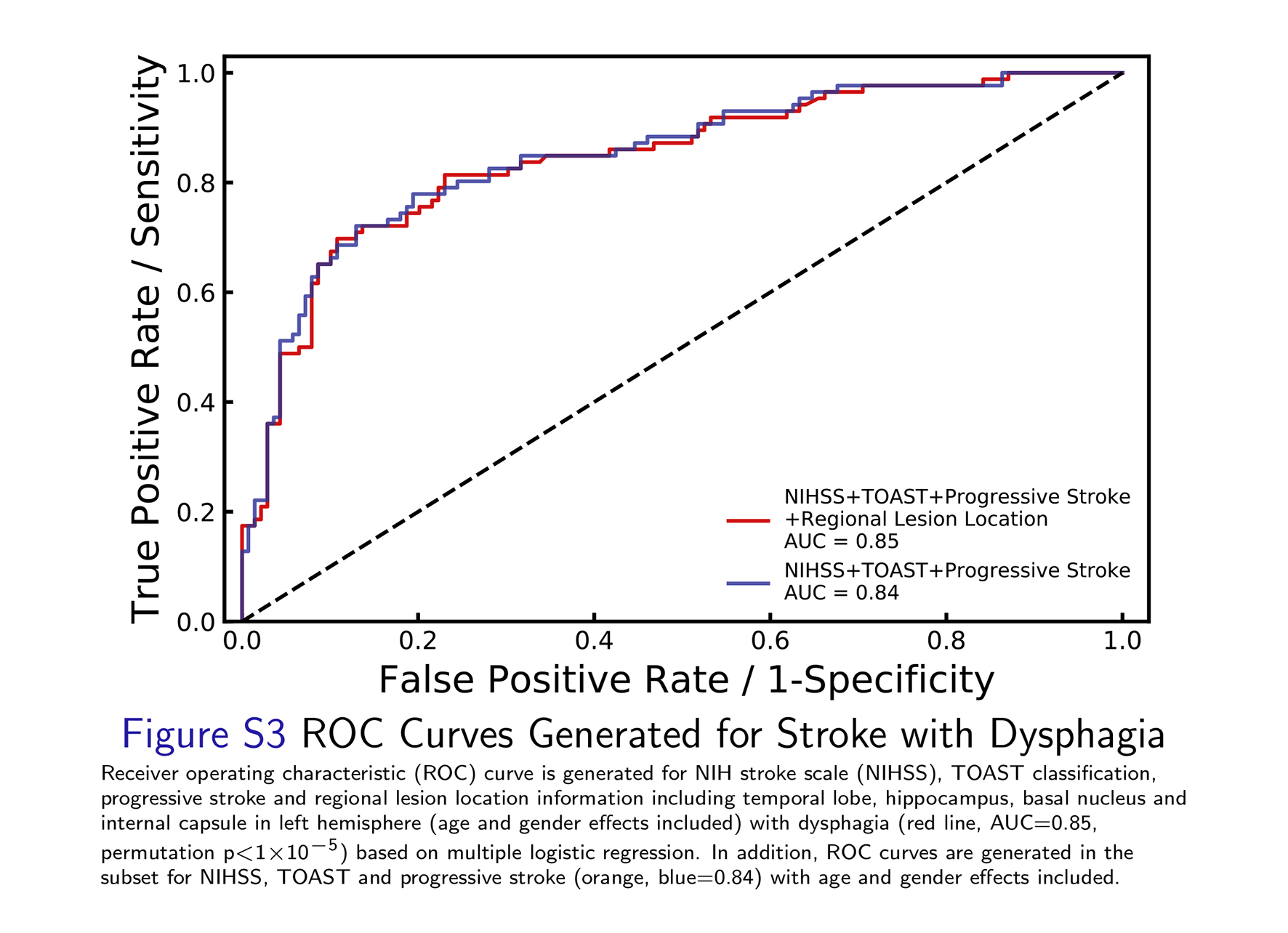

Supplement: Supplementary Figure 3 — ROC curves generated for stroke with dysphagia. Receiver operating characteristic (ROC) curve is generated for NIH stroke scale (NIHSS), TOAST classification, progressive stroke and regional lesion location (age and gender effects included) with dysphagia (red line, AUC = 0.85, permutation p < 1 × 10^–5^) based on multiple logistic regression. ROC curves are generated for NIHSS, TOAST classification and progressive stroke (blue, AUC = 0.84) with age and gender effects included. [file Image_3.tif]
